# Korea BioData Station (K-BDS)

**DOI:** 10.5808/gi.21.1.e1

**Published:** 2023-03-31

**Authors:** Taesung Park

**Affiliations:** Department of Statistics, Seoul National University, Seoul 08826, Korea

This issue contains an important article regarding a biological data repository in Korea by Lee et al. (Korea Bioinformation Center, KOBIC, Korea). Lee and his colleagues introduced a new biological data repository, the Korea BioData Station (K-BDS) for sharing biological data (https://www.kbds.re.kr). The term “biological data” refers to information derived from living organisms and their products. The unique characteristics of biological data make biological data management particularly challenging. Diverse biological research generates vast amounts of different types of big data, such as sequence data and protein structures, in a variety of research fields, including transcriptomics, genomics, proteomics, and metabolomics. The scale of biological data is growing exponentially due to rapid technological advances and declining prices for new technologies used to produce biological data. Biological information now plays an essential role in scientific progress. As such, it is very complex compared to other forms of data. The integrated analysis of different data types is attracting research interest these days.

There are many biological databases around the world, such as the International Nucleotide Sequence Database Collaboration (INSDC), GenBank, Protein Data Bank (PDB), and DNA Data Bank of Japan (DDBJ). These databases were built to share vast biological datasets and have played an important role in scientific research by storing, managing, and providing access to increasingly large datasets. The future of biological research is highly dependent on the proper use and management of data. However, the complexity of biological data and the unprecedented exponential rate of production present significant challenges in archiving, integrating, and translating big data.

Despite the government's large-scale R&D investments in the field of biology, Korea has not previously taken active steps toward the establishment of a biological database. Recently, however, the Korean government officially announced a national strategy for sharing biological data generated with national R&D funding. The Korean government has funded the Korea Bioinformation Center (KOBIC) to develop a new data repository, the Korea Biodata Station (K-BDS).

The K-BDS is a data repository for all types of data for biological research. It provides free access to various database resources to archive raw biological data and support research activities. The data structure of K-BDS uses international data standards and formats. The K-BDS will evolve through the optimization and automation of data submission, curation and analysis procedures, infrastructure upgrades for storing big data, and the migration and development of new tools and pipelines to support biological research. I hope that the K-BDS will become a centralized archive in Korea and support research activities in academia and industry around the world.

